# Impact of White Bread Fortified With Carob Molasses Pulp Flour and Wheat Germ on Glycemic Response and Antioxidant Capacity in Healthy Subjects

**DOI:** 10.1002/fsn3.71046

**Published:** 2025-12-04

**Authors:** Ebrar Tuşat, Eda Parlak, Gonca Yıldırım, Kamuran Öztop, Betül Gülşen, Yüksel Özdemir, Yücel Uysal

**Affiliations:** ^1^ Department of Nutrition and Dietetics, Faculty of Health Sciences Toros University Mersin Turkey; ^2^ Department of Hotel Restaurant and Catering Services Vocational School, Toros University Mersin Turkey; ^3^ Department of Family Medicine Faculty of Medicine, Mersin University Mersin Turkey

**Keywords:** dietary fiber, functional foods, nutritional intervention, polyphenols, postprandial glucose

## Abstract

This clinical trial aimed to develop a functional white bread formulation enriched with carob molasses pulp (CMP) flour and wheat germ—nutrient‐rich by‐products with high insoluble fiber and antioxidant content—and to evaluate its effects on glycemic response and antioxidant capacity in healthy individuals. In the formulation phase, five bread samples with 0%–20% CMP flour were developed. Based on sensory scores and fiber/starch composition, wheat germ (0%–20%) was incorporated into selected CMP breads to optimize acceptability and nutritional quality. The final test bread (20% CMP +20% wheat germ) and control bread (white bread) underwent physical and chemical analyses, including dietary fiber, resistant starch, and antioxidant capacity. In the intervention phase, 16 healthy volunteers (aged 19–35) consumed each bread for 1 week. Blood glucose (BG), total antioxidant status (TAS), total oxidant status (TOS), and Visual Analogue Scales (VAS) satiety and appetite scores were evaluated at fasting and at 30, 60, 120, and 180 min post‐consumption. Compared to control bread, the test bread had significantly higher fiber, polyphenols, antioxidants, and resistant starch (*p* < 0.001). No significant differences were observed in BG, TAS, or TOS (*p* > 0.05). However, VAS results showed that the test bread enhanced satiety, particularly at 0 and 30 min (*p* < 0.001). In conclusion, a sensory‐acceptable, functional bread was developed. While it did not significantly alter glycemic or oxidative responses in the short term, it may contribute to appetite regulation.

**Trial Registration:** ClinicalTrials.gov identifier: NCT06200272

## Graphical abstract

Graphical abstract illustrating the formulation steps and enhanced physical and chemical properties of breads enriched with carob molasses pulp flour and wheat germ compared to control bread.

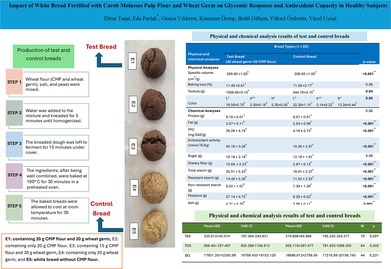

## 1 Introduction

Lifestyle factors, particularly diet, play a critical role in the development and progression of diseases associated with oxidative stress. A diet rich in antioxidants and polyphenols helps maintain redox balance, reduce oxidative damage, and support overall health and longevity (Frank et al. 2021; Küçükgül 2016). Measurements of TAS and TOS provide important insights into the body's oxidative balance; low TAS and high TOS levels have been associated with conditions such as cardiovascular diseases, cancer, neurodegenerative disorders, and aging (Çebi and Gülçeri 2023).

Bread is a staple food widely consumed around the world and an important source of nutrition. The average annual per capita consumption of bread worldwide is approximately 70 kg, with an average of 59 kg/year in Europe. The lowest consumption is observed in the United Kingdom and Italy (approximately 31 kg/year), while the highest consumption is in Bulgaria (95 kg/year) (Eglite and Kunkulberga 2017; De Boni et al. 2019). In Turkey, the average daily bread consumption is high, reported as 179.8 ± 130.39 g/day (TNHS 2019). Given its widespread consumption, the nutritional quality of bread can have significant effects on public health.

Baked goods with a high glycemic index (GI) cause rapid increases in blood glucose (BG) levels, contributing to insulin resistance, weight gain, inflammation and an increased risk of type 2 diabetes. Milling processes typically remove wheat bran, which reduces fiber content while increasing glycemic index and glycemic load and lowering antioxidant content (Shafi et al. 2017; Borczak et al. 2018). Therefore, enriching bread with functional ingredients rich in fiber and antioxidants is an important strategy for reducing the risk of chronic diseases.

Carob pulp is a by‐product of carob molasses production and is rich in fiber and biologically active compounds; it provides benefits through its glycemic control and antioxidant effects (Turhan and Karhan 2004; Macho‐González et al. 2017). Wheat germ is also a by‐product lost during milling and contains important components that support antioxidant defense and contribute to overall health, such as tocopherols, sterols, carotenoids, and glutathione (Boukid et al. 2018). Although various studies have been conducted on bread enriched with carob flour (Martić et al. 2022; Issaoui et al. 2021; Novotni et al. 2020), there is no study investigating the effects of breads made with a combination of CMP flour and wheat germ on glycemic response and plasma antioxidant capacity in healthy individuals.

The evaluation of by‐products generated in food production has gained importance with the increasing interest in reducing food waste and utilizing secondary resources (Teixeira et al. 2014). Therefore, in this study, white bread formulations enriched with CMP flour and wheat germ in terms of fiber and antioxidants were developed; their sensory properties were evaluated, and the effects of the developed breads on postprandial glycemic response and antioxidant capacity in healthy individuals were investigated.

## 2 Materials and Methods

### 2.1 Study Design

This study was a randomized, single‐blind, crossover controlled experimental study. The study consisted of two phases. In the first phase, bread production and the physical and chemical analyses were conducted between August and December 2023, with three parallels and two repetitions. As the control bread, white bread produced with wheat flour without the addition of CMP flour and wheat germ was used. As the test bread, a formulation that included CMP flour and wheat germ was used. The second phase, during which the test and control breads were consumed by healthy individuals, was carried out in January 2024.

### 2.2 Phase 1

#### 2.2.1 Bread Production

Initially, bread formulations containing varying proportions of CMP flour were prepared. The CMP used in bread production was obtained from a company that produces carob molasses. While obtaining CMP flour, after the wet pulp residue remaining after carob molasses production was first dried at room temperature for 10 days, it was dried in an oven at 120°C for 4 h in portions sized 2–3 cm, then left to equilibrate for 30 min in a desiccator and transferred to airtight, moisture‐impermeable sealed storage bags. After the dried CMP residue was milled using a grinder, it was sieved through a 100 μm mesh sieve and CMP flour was obtained from the fraction with particle sizes under 100 μm.

Common ingredients such as salt, water, and yeast were used in all bread samples, and both test and control breads were produced using the same method. The wheat flour, salt, yeast, and wheat germ used in bread production were purchased from a local market on the day of production and stored in conditions that prevented exposure to heat, light, and humidity. Yeast and CMP were stored in sealed bags at +4°C in a refrigerator to prevent moisture absorption.

**FIGURE 1:**
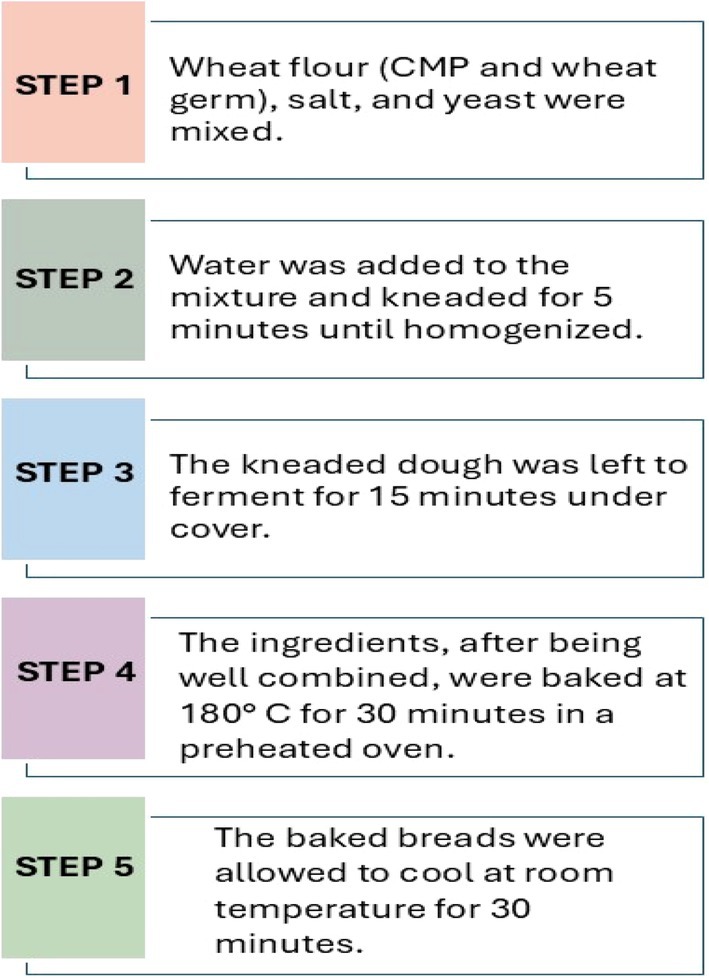
Production of test and control breads.

For the control bread production, wheat flour (100 g), salt (1.5 g), and yeast (4 g) were mixed until they became homogeneous; then water (60 mL) was added. The same fixed amount of water was used in both the control and experimental formulations, and all other non‐flour ingredients were standardized. The amount of water was not adjusted based on differences in flour composition, thereby minimizing external variability and more clearly demonstrating the effects of flour composition. In samples supplemented with CMP flour, the amount of wheat flour was reduced by the quantity of added CMP flour. On the other hand, for breads enriched with wheat germ, the germ was added additionally to the formulation. The final formulations have been adjusted to ensure that the dry matter content of the breads given to participants is equal; thus, 50 g portions correspond to an equal amount of solid matter in all varieties. The production phases of the test and control breads are illustrated in Figure 1.

#### 2.2.2 Decision‐Making Phase of the Test Bread and Sensory Analyses

Five different bread formulations were developed by adding wheat flour with CMP flour at levels of 0%, 5%, 10%, 15%, and 20% as a substitute for the amount of wheat flour used in the white bread formulation. Samples for wheat germ addition were selected based on sensory analysis results and their glycemic response potential. The glycemic response potential was evaluated without direct measurement of the glycemic index, considering the total starch, resistant starch, and dietary fiber contents.

Sensory analysis was conducted by a trained panel consisting of 20 academic staff members from Toros University using a 7‐point Hedonic Scale. The evaluators assessed the bread samples using the 7‐point hedonic test, rating them on a scale from 1 (dislike extremely) to 7 (like extremely). Panelists were also given water to rinse their mouths before and after tasting each sample. Bread samples receiving a score of 5 or above in the overall evaluation were considered liked and acceptable. The panelists evaluated the breads based on criteria including appearance, softness, crumb structure, stickiness, color, taste, firmness, and overall acceptability. The sensory analyses were repeated three times (Ekici et al. 2015).

In addition to the three bread samples containing acceptable levels of CMP flour identified in the sensory analysis, bread formulations were prepared by adding wheat germ at levels of 0%, 5%, 10%, 15%, and 20%. Sensory analysis of these formulations was conducted by a trained panel of 20 academic staff using a 7‐point Hedonic Scale. The results were evaluated in the same manner as applied to the CMP flour‐containing bread formulations (Ekici et al. 2015). The final test bread formulation was determined by considering both the nutritional content and the sensory analysis outcomes.

#### 2.2.3 Physical and Chemical Analysis Methods Applied to the Bread Samples

For the purpose of conducting physical and chemical analyses on the bread produced with the selected test formulation and the control bread, the bread samples were dried in an oven at 105°C for 4 h. The dried bread samples were then ground into fine particles using a grinder and the analyses were performed. For the CMP flour added to the test bread, no additional treatment was applied except for the previously conducted processing steps, and analyses were conducted directly. All chemical analyses were performed on a 100 g basis in accordance with standard reporting practices, and the bread portion given to participants for consumption in Phase 2 was 50 g.

##### 2.2.3.1 Physical Analyses

###### 2.2.3.1.1 Determination of Baking Loss

Baking loss was calculated based on the difference between the weight of the dough before baking and the weight of the cooled bread after baking. The calculation was performed using the following formula (Barışık and Tavman 2018):

$$\text{Baking Loss}\;\left(\%\right)=\left[\left(\text{Initial Dough Weight}-\text{Baked Bread Weight}\right)/\text{Initial Dough Weight}\right]\times 100$$

###### 2.2.3.1.2 Determination of Specific Volume

The volumes of the breads were determined using the flaxseed displacement method according to the AACC 10–05.01 method. Samples were measured after they were rested at room temperature for 4 h (Yıldız et al. 2021).

###### 2.2.3.1.3 Color Determination

The crumb color of the bread was measured using a colorimeter (PCE‐CSM 1), and the L* (lightness), a* (green‐red), and b* (blue‐yellow) values were determined. Measurements were taken from three different points within the bread crumb (Özdemir et al. 2021).

###### 2.2.3.1.4 Texture Analysis

The firmness of bread samples was measured using a Texture Analyzer according to the AACC 74–09 method. The measurements were performed on the crust surface of samples rested at 25°C for 16 h after baking (Aydın et al. 2022). In this study, hardness was evaluated specifically in bread crusts, as the focus of the research was on the mechanical properties of the crust, which are directly affected by baking conditions and formulation. The evaluation of crust hardness provides valuable information about the structural integrity, sensory properties, and potential shelf life of bread, which is why it was chosen as the parameter of interest in this study.

##### 2.2.3.2 Chemical Analyses

###### 2.2.3.2.1 Moisture Determination

Moisture content was determined by oven drying at 105°C according to the AOAC 925.10 method (Pojić et al. 2015).

###### 2.2.3.2.2 Ash Determination

Ash determination was conducted by incineration in a muffle furnace at 550°C according to the AOAC 923.03 method (Pojić et al. 2015).

###### 2.2.3.2.3 Fat Determination

Fat content was determined using the Soxhlet extraction method (López‐Bascón and De Castro 2020). The amount of fat extracted with petroleum ether solvent was measured gravimetrically (Büyüktuncel 2012).

###### 2.2.3.2.4 Protein Determination

Protein content was determined by measuring the nitrogen content using the Kjeldahl method. The percentage of nitrogen was converted to the percentage of protein by multiplying by a factor of 6.25 (Abrams et al. 2014).

###### 2.2.3.2.5 Total Phenolic Content Determination

Total phenolic content was spectrophotometrically determined using the Folin–Ciocalteu method. Measurement was taken at 650 nm, and results were reported as tannic acid equivalents (Singleton et al. 1999).

###### 2.2.3.2.6 Determination of Antioxidant Content

The DPPH radical scavenging method was used, and absorbance measurements were measured at 515 nm. Results were calculated as mmol Trolox equivalents per kilogram (Brand‐Williams et al. 1995; Mogalhaes et al. 2006).

###### 2.2.3.2.7 Total Sugar Determination

Total sugar content was determined using the phenol‐sulfuric acid method, and absorbance measurements were taken at 490 nm (Özdemir et al. 2022).

###### 2.2.3.2.8 Total Dietary Fiber Determination

Total dietary fiber was determined gravimetrically after acid hydrolysis and solvent extraction. Samples were dried in an oven at 105°C and they were kept in a desiccator until constant weight was achieved (Keçeli 2016).

###### 2.2.3.2.9 Total Starch and Resistant Starch Determination

Total starch and resistant starch were determined using the Megazyme K‐RSTAR kit in accordance with AOAC 2002.02, AACC 32‐40.01, and Codex Type 2 methods. Absorbance measurements were taken at 510 nm. Results were expressed as percentages (Li et al. 2021; Kaur et al. 2023).

###### 2.2.3.2.10 Digestible Carbonhydrate and Energy Content

The digestible carbohydrate content was calculated by subtracting resistant starch from total starch and adding the sugar content. The energy content was estimated using Atwater factors: 4 kcal/g for protein and carbohydrates, 9 kcal/g for fat, and 2 kcal/g for dietary fiber (Food and Agriculture Organization (FAO) 2003).

### 2.3 Phase 2

#### 2.3.1 Participants

The study was conducted with the participation of 16 healthy and voluntary individuals aged between 19 and 35 years. Inclusion criteria consisted of no use of prescription medications or dietary supplements, not being pregnant or lactating, non‐smoking, abstaining from alcohol or consuming it moderately (< 2 drinks/day), having a Body Mass Index (BMI) between 18.5 and 24.9 kg/m^2^, absence of gluten intolerance and insulin resistance, no history of food allergies, and signing the informed consent form. Participants' health status was assessed through physical examinations and biochemical tests conducted by a specialist physician. Individuals who did not meet the specified criteria were not included in the study.

All procedures performed in studies involving human participants were in accordance with the ethical standards of the institutional and/or national research committee and with the 1964 Helsinki declaration and its later amendments or comparable ethical standards. For this study, approval was obtained from the Mersin University Clinical Research Ethics Committee on August 17, 2022 (number: 2022/562), and written informed consent was obtained from all participants before the study. This study was registered at ClinicalTrials.gov. In this study, for determining the sample size, a power analysis was performed with G*Power 3.1 software using alpha (*α*) = 0.05, power (1 − *β*) = 0.95, and *d* = 1.75 (medium effect size), which indicated that at least 6 individuals were required per group (Kang 2021). To anticipate potential dropouts (withdrawal from the study or irregular attendance), it was decided to recruit 20 volunteers. Since two participants withdrew from the study and another two were excluded due to non‐compliance with study timing, the study was completed with 16 volunteers.

#### 2.3.2 Data Collection

In the study, healthy participants were provided to consume a 50 g portion of either the test bread (bread enriched with CMP flour and wheat germ) or the control bread (white bread) in a single‐blind randomized controlled crossover design in the first week and the other in the second week. The portion size was the same for all types of bread, and participants were asked to consume the entire portion they were given. The breads were consumed alone, without any other foods or drinks, and participants ate the breads individually and separately from each other in order to prevent any possible social or behavioral effects. The portion size (50 g) was determined in accordance with the portion definition specified for bread in the Food‐Based Dietary Guidelines for Turkey (Ministry of Health, Republic of Turkey 2016) (1 medium slice ≈ 50 g) and is also consistent with previous sensory evaluation studies conducted on bread (Heenan et al. 2008). This amount was selected to be sufficient for evaluation without causing satiety.

The data collection tools used in the study included: a sociodemographic information form, dietary intake, anthropometric measurements, Visual Analog Scale (VAS), and biochemical parameters. Sociodemographic characteristics and anthropometric measurements of the healthy individuals were recorded once at the beginning of the study; their dietary intake records were collected over 2 days in total, with 1 day recorded in the first week and 1 day in the second week. Visual Analog Scale (VAS) values and the biochemical parameter BG were recorded a total of 10 times, 5 times in the 1st and 2nd weeks of the study, at the following time points: 0 min (after 12 h of overnight fasting), and at 30, 60, 120, and 180 min after the consumption of the test/control bread. Among the biochemical parameters, TAS and TOS values were recorded a total of 6 times, 3 times in the 1st and 2nd weeks of the study, at the following time points: 0 min (after 12 h of overnight fasting), and at 60 and 180 min after the consumption of the test/control bread.

##### 2.3.2.1 Sociodemographic Information

At the beginning of the study, the participants' gender, age, health status, education level, and medication use were queried by the research dietitian and recorded in the form.

##### 2.3.2.2 Dietary Intake

Dietary intake records of the healthy individuals were collected by the research dietitian using a 24‐h dietary recall form on 1 day during the 1st week and 1 day during the 2nd week, totaling 2 days. These records included all foods and beverages consumed within the 24 h immediately before the consumption of the test or control bread. Food consumption data were obtained through in‐person interviews with the healthy individuals, using portion sizes (g, mL) and quantities, following the five‐step multiple‐pass recall method by the Food and Agriculture Organization (FAO) (2018).

##### 2.3.2.3 Anthropometric Measurements

Anthropometric measurements were performed by the research dietitian at the beginning of the study, and the data were recorded on the designated forms.

###### 2.3.2.3.1 Body Composition

Body compositions of the healthy individuals were assessed using Bioelectrical Impedance Analysis (BIA) with the Tanita BC‐418 body composition analyzer. The device recorded participants' body weight, body fat percentage (%), fat mass (kg), muscle mass (kg), and total body water (TBW) mass (kg). The analysis results were evaluated by the research dietitian. Body Mass Index values were calculated using the formula body weight (kg) divided by the square of height (m^2^) and interpreted based on the World Health Organisation (WHO) (2010) reference values for healthy individuals. Body fat percentage was considered normal within the ranges of 15%–18% for males and 22%–27% for females (Dinesh et al. 2022).

###### 2.3.2.3.2 Height Measurement

Height measurements of the healthy individuals were evaluated using an infrared stadiometer. The device was fixed to the wall, and measurements were taken with the participants standing with their feet together and head positioned in the frankfort horizontal plane (aligning the infraorbital margin and the upper margin of the external auditory meatus) (Dinesh et al. 2022).

###### 2.3.2.3.3 Circumference Measurements

Waist (cm) and hip circumferences (cm) of healthy individuals were measured using a non‐stretchable measuring tape, and waist‐to‐hip ratios were calculated (Chinedu et al. 2013). The waist and hip measurements were evaluated based on the World Health Organisation (WHO) (2008) recommended healthy reference values (waist circumference: < 80 cm for women, < 94 cm for men; waist‐to‐hip ratio: < 0.85 for women, < 0.90 for men).

##### 2.3.2.4 Visual Analog Scales

The Visual Analog Scale questionnaire consists of a satiety scoring table (hunger, fullness, desire to eat, and prospective consumption) and an appetite scoring table for certain tastes (sweet, salty, sour, and fatty), and they are questioned by a scoring system. Responses related to each question in the VAS questionnaire are measured on a 10 cm horizontal line with fixed endpoints. Participants rate their current sensation intensity by marking a point between 1 and 10. VAS scores were collected by the researcher dietitian from participants a total of 10 times during both the 1st and 2nd weeks, at 0, 30, 60, 120, and 180 min.

During scoring, the scales were interpreted as follows: *Hunger*: 1: “not at all hungry”; 10: “hungrier than ever before”; *desire to eat/satiety*: 1: “my stomach is completely empty”; 10: “I cannot eat another bite”; *fullness*: 1: “not full at all”; 10: “very full”; and *prospective consumption*: 1: “I cannot eat anything”; 10: “I can eat much more.” The Overall Appetite score was calculated using the formula: (hunger score + desire to eat/satiety score + [100 − fullness score] + prospective consumption score)/4 (Vuksan et al. 2009, 2016).

In the second part of the VAS questionnaire, it was scored as follows: *the desire to eat sweet foods*: 1 = “yes, I want it very much”; 10 = “no, I do not want it at all”;*the desire to eat salty foods*: 1 = “yes, I want it very much”; 10 = “no, I do not want it at all”; *the desire to eat sour foods*: 1 = “yes, I want it very much”; 10 = “no, I do not want it at all”; and *the desire to eat fatty foods*: 1 = “yes, I want it very much”; 10 = “no, I do not want it at all”. These scores are evaluated in the opposite manner compared to the scoring for the thought of hunger, fullness, desire to eat, and prospective consumption (Akkuş et al. 2023).

##### 2.3.2.5 Biochemical Parameters

###### 2.3.2.5.1 BG Measurements

Venous BG levels were measured at 0 (fasting), 30, 60, 120, and 180 min during both study weeks (test and control bread consumption weeks) for healthy individuals. Measurements were conducted using a calibrated glucometer, with each reading taken twice and the average value considered for analysis. Additionally, venous blood samples were collected by authorized healthcare personnel, and analyses were performed by qualified laboratory specialists. Blood glucose levels were analyzed using a Hitachi 912 autoanalyzer with Roche Diagnostics kits (Roche Diagnostics, Indianapolis, USA). Internal quality control procedures were applied in the laboratory analyses. Reference values were set as fasting BG (FBG) (0 min) < 100 mg/dL and 120‐min postprandial BG (PBG) (120 min) < 140 mg/dL (ElSayed et al. 2023).

###### 2.3.2.5.2 Total Antioxidant Status (TAS) and Total Oxidant Status (TOS) Measurements

During both study weeks, participants' serum TAS and TOS levels were evaluated at 0 (baseline), 60, and 180 min. Venous blood samples were collected by authorized healthcare personnel, and serum aliquots were transported to the R&D unit of a specialized laboratory on dry ice within 10 min for analysis. When necessary, samples were stored according to protocol to maintain stability: up to 1 week at +4°C, 6 months at −20°C, and 1 year at −80°C. TAS levels were determined using commercial kits (Rel Assay Diagnostics, Turkey) based on the reduction of ABTS radical to a colorless form by antioxidants. Measurements were performed with a spectrophotometer (Biochrom Libra S22) at 660 nm, and results were expressed as mmol Trolox equivalent per liter (mmol Trolox Eq/L) (Erel 2005). TOS levels were determined using a method relying on the oxidation of ferrous ion‐chelator complex to ferric ions and their reaction with a chromogen, also employing the Rel Assay Diagnostics kit. Measurements were taken at 530 nm, and results were reported as μmol hydrogen peroxide equivalent per liter (μmol H_2_O_2_ Eq/L) (Erel 2005). Reference ranges were set as 1.20–1.50 mmol/L for TAS and 4.00–6.00 μmol/L for TOS (Özgün et al. 2013).

### 2.4 Statistical Analyses

The demographic characteristics of the participants were summarized using descriptive statistics (frequency, percentage, mean, standard deviation). The physical and chemical properties of the test and control breads were compared using the independent samples *t*‐test. Changes in nutrient intakes were analyzed based on median values using the Wilcoxon signed‐rank test. Differences in Visual Analog Scale (VAS) scores related to test and control bread consumption were examined with the Friedman test. For biochemical parameters (TAS and TOS: 0, 60, and 180 min; BG: 0, 30, 60, 120, and 180 min), area under the curve (AUC) values were calculated using the trapezoidal method to evaluate changes over time. Repeated measurement AUC values were compared between bread types using the Wilcoxon signed‐rank test, and results were reported as *W* and *p* values. All statistical analyses were conducted using IBM SPSS Statistics (Version 26.0) and R software (Version 4.5.1; R Foundation for Statistical Computing, Vienna, Austria). A significance level of *p* < 0.05 was accepted.

## 3 Results

### 3.1 The Determination of the Test Bread Formulation and Sensory Analysis

At the beginning of the study, sensory analysis was conducted on breads with added CMP flour as a wheat flour substitute at levels of 0%, 5%, 10%, 15%, and 20%. Breads receiving an Overall Appetite score of ≥ 4 were considered acceptable for consumption and based on these results, the samples with 0%, 15%, and 20% CMP flour addition were evaluated as acceptable. According to the sensory analysis results, breads containing 0%, 5%, 10%, 15%, and 20% wheat germ additions to the 0%, 15%, and 20% CMP flour‐enriched samples which received an Overall Appetite score of ≥ 4 were also considered acceptable for consumption.

**FIGURE 2:**
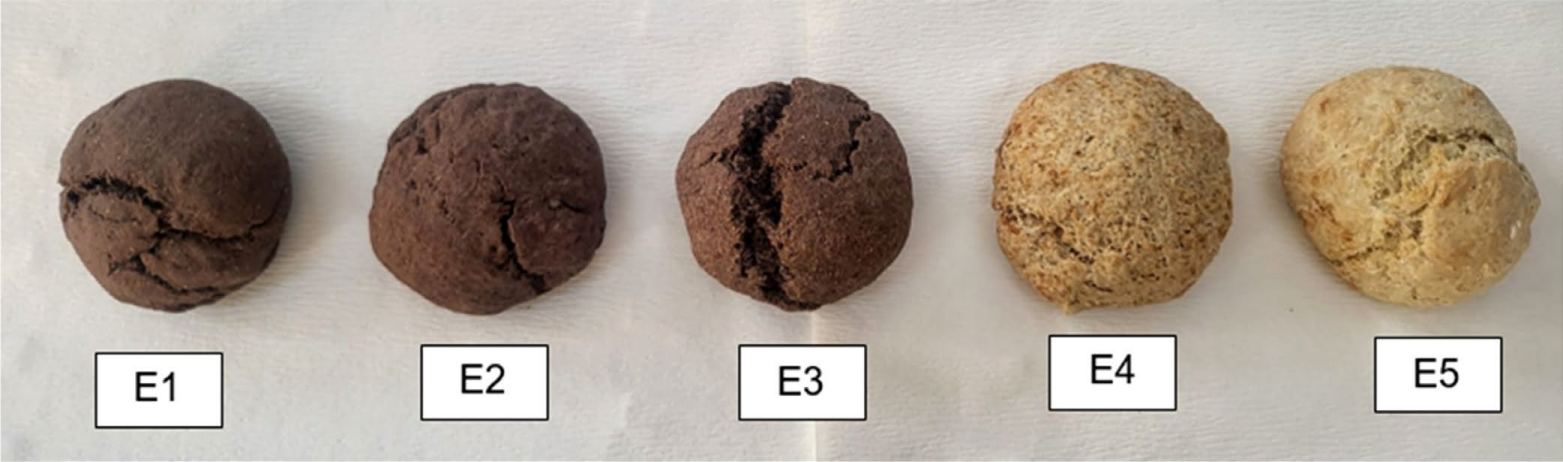
Bread formulations containing different amounts of CMP flour and wheat germ (from left to right: E1: Containing 20 g CMP flour and 20 g wheat germ, E2: Containing only 20 g CMP flour, E3: Containing 15 g CMP flour and 30 g wheat germ, E4: Containing only 20 g wheat germ, and E5: White bread without CMP flour).

According to the sensory analysis evaluation results, bread formulations containing only 20 g CMP flour only 20 g wheat germ, 20 g CMP flour combined with 20 g wheat germ, and 15 g CMP flour combined with 30 g wheat germ were accepted in terms of consumability (Figure 2). Among the breads that scored statistically higher than the control bread (E5) in the overall acceptability parameter of the sensory analysis, the bread with the richest content was accepted as the test bread (E1) (Table 1). Based on the overall evaluation, the test bread formulation (20 g CMP flour +20 g wheat germ), which received statistically significantly higher scores than the control bread in taste and aroma, texture, and overall acceptability parameters, was selected as the final test bread due to its superior nutritional value and sensory properties.

**TABLE 1:** Sensory analysis parameters of different bread types.

Sensory analysis	Bread types (x̄ ± SD)					*p*
	E1 (20 wheat germ + 20 CMP flour)	E2 (20 CMP flour)	E3 (20 wheat germ + 15 CMP flour)	E4 (20 wheat germ)	E5 (control)	
Color	4.90 ± 1.00^a.b^	4.90 ± 1.16^a.b^	4.95 ± 1.05^a^	4.85 ± 1.18^a.b^	4.65 ± 1.18^b^	**0.048** *
Internal color	4.85 ± 1.05^a^	5.00 ± 1.41^a^	4.80 ± 1.24^a.b^	4.60 ± 1.23^b^	4.25 ± 1.21^b^	**0.037** *
Taste ve Aroma	5.10 ± 1.20^a^	4.30 ± 1.38^b^	3.90 ± 1.59^c^	4.70 ± 1.17^b^	4.65 **±** 1.46^b^	**< 0.001** **
Texture	4.70 ± 1.00^a^	4.85 ± 1.35^a^	4.25 ± 1.37^b^	4.40 ± 1.19^a.b^	4.05 ± 1.43^c^	**0.025** *
Chewiness	4.80 ± 1.05^a^	4.90 ± 1.37^a^	4.70 ± 1.17^a.b^	4.20 ± 1.47^b^	4.40 ± 1.60^b^	0.063
Hardness	4.75 ± 1.15^a^	5.30 ± 1.08^a^	4.70 ± 0.98^a^	4.00 ± 1.68^a^	4.40 ± 1.31^b^	0.071
Crust hardness	4.90 ± 1.05^a^	5.15 ± 0.99^a^	4.60 ± 0.94^a^	4.25 ± 1.71^a^	4.75 ± 1.37^b^	0.085
Overall acceptability	5.10 ± 1.00^a^	4.80 ± 1.32^a.b^	4.15 ± 1.31^b^	4.45 ± 1.36^b^	4.50 ± 1.36^b^	**0.033** *

*Note:* Different superscript letters in the same row indicate statistically significant differences (ANOVA, Duncan post hoc test).

Abbreviations: x̄, mean; CMP, carob molasses pulp; SD, standard deviation.

* *p* < 0.05.

** *p* < 0.001.

### 3.2 The Physical and Chemical Analyses of Bread Types

The physical and chemical analyses of the test and control breads that were sensorially accepted were examined, and the results of the test and control bread formulations consumed only by healthy individuals are presented in Table 2. Among the physical analyses of the test bread, its specific volume (280.00 ± 1.00) was found to be higher than that of the control bread (200.00 ± 1.00), and the difference between them was found to be significant (*p* < 0.001). Regarding color parameters, the test bread showed significantly lower L* (lightness), a* (redness), and b* (yellowness) values compared to the control bread (*p* = 0.05). This indicates that the additive used in the test bread exerted a darkening effect on the color.

**TABLE 2:** Physical and chemical analysis results of test and control breads.

Physical and chemical analyses	Bread types (x̄ ± SD)						*p*
	Test bread (20 wheat germ + 20 CMP flour)			Control bread			
*Physical analyses*							
Spesific volüme (cm^3^/g)	280.00 ± 1.00^b^			200.00 ± 1.00^a^			< 0.001**
Baking loss (%)	11.49 ± 0.61^a^			11.58 ± 2.17^a^			0.06
Texture (g)	1009.90 ± 0.10^b^			944.70 ± 0.10^a^			**0.04**
Color	L*	a**	b*	L*	a**	b*	**0.05**
	16.50 ± 0.75^b^	2.59 ± 0.18^b^	5.35 ± 0.30^b^	32.26 ± 1.10^a^	3.14 ± 0.22^a^	13.34 ± 0.44^a^	
*Chemical analyses*							
Protein (g)	9.18 ± 0.01^a^			9.07 ± 0.01^a^			0.06
Fat (g)	3.57 ± 0.11^b^			2.54 ± 0.08^a^			< 0.001**
TPC (mg GAE/g)	26.28 ± 4.74^b^			4.18 **±** 0.74^a^			< 0.001**
Antioksidant activity (mmol TE/kg)	40.18 ± 3.28^b^			18.36 ± 2.97^a^			< 0.001**
Sugar (g)	10.19 ± 2.19^a^			12.18 ± 1.91^a^			0.08
Dietary fiber (g)	10.64 ± 2.23^b^			2.97 ± 0.12^a^			< 0.001**
Total starch (g)	20.51 ± 5.33^b^			18.91 ± 2.37^a^			< 0.001**
Resistant starch (g)	14.49 ± 3.26^b^			11.52 ± 2.53^a^			< 0.001**
Non‐resistant starch (g)	6.02 ± 1.07^b^			7.39 ± 1.39^a^			< 0.001**
Moisture (g)	37.14 ± 4.73^b^			9.29 ± 0.03^a^			< 0.001**
Ash (g)	4.31 ± 0.30^b^			3.98 ± 0.11^a^			0.001**

*Note:* Indepented sample *t*‐test: *p < 0.05; **p < 0.001.

Abbreviations: x̄, mean; CMP, carob molasses pulp; GAE, gallic acid equivalent; SD, standard deviation; TE, Trolox equivalent; TPC, total phenolic compounds.

**FIGURE 3:**
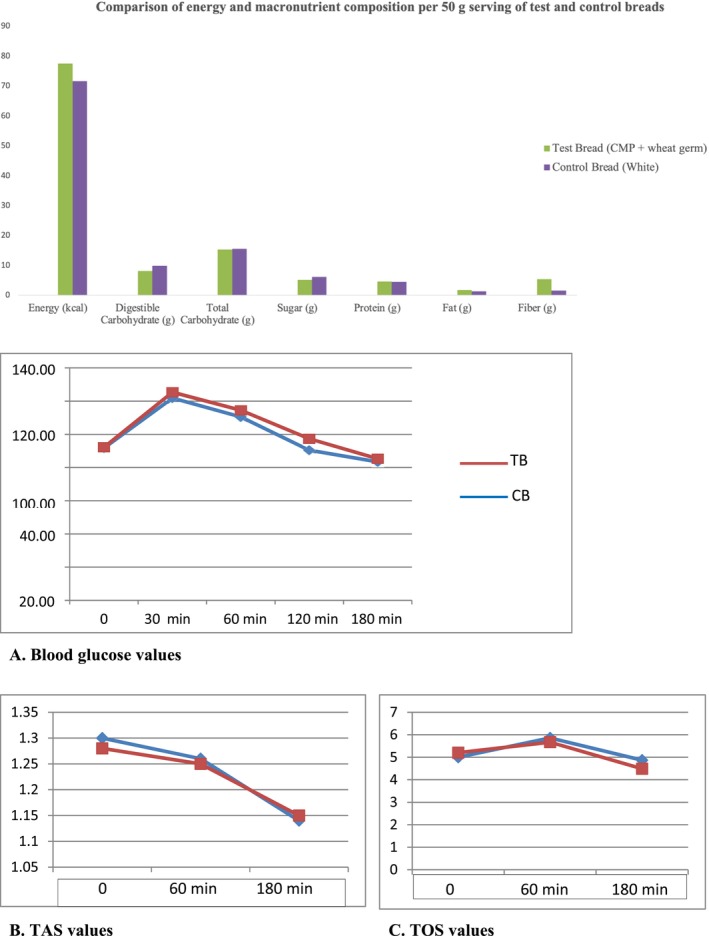
Energy and macronutrient composition per 50 g serving of test and control breads. (A–C) Comparison of biochemical parameters between test and control bread. BG, blood glucose; CB, control bread; min, minutes; TAS, total antioxidant status; TB, test bread; TOS, total oxidant status.

Chemical analyses of the test bread revealed significantly higher contents of fat, total polyphenols (TPC), antioxidants, total starch, resistant starch, moisture, and ash compared to the control bread (*p* < 0.001). The dietary fiber content in the test bread was also significantly higher than in the control bread (with values of 10.64 ± 2.23 g and 2.97 ± 0.12 g, respectively) (*p* < 0.001) (Table 2). The energy and macronutrient contents corresponding to the 50 g portions consumed by the participants are presented in Figure 3. The test bread contains slightly higher fiber and fat, but lower digestible carbohydrates compared to the control bread. However, the overall differences in energy and macronutrient composition are small, and given the nature of the study design, it is unlikely that these differences would significantly affect the observed glycemic or appetite responses.

### 3.3 Healthy Individuals

#### 3.3.1 Sociodemographic Characteristics and Anthropometric Measurements

The mean age of the healthy individuals included in the study was found to be 25.00 ± 2.28 years; 56.3% (*n* = 9) of the participants were female and 43.8% (*n* = 7) were male. The sociodemographic characteristics and anthropometric measurements of the healthy individuals are presented in Table 3.

**TABLE 3:** Sociodemographic characteristics and anthropometric measurements of healthy individuals.

Variables	x̄±SD	Range (min–max)
Height (cm)	171.56 ± 10.00	155.00–188.00
Body weight (kg)	65.72 ± 11.52	48.50–85.70
BMI kg/m^2^	22.12 ± 1.96	18.50–25.90
BMR (kkal)	1491.63 ± 280.45	1058.00–1918.00
Body fat percentage %	21.84 ± 7.34	9.20–34.50
Fat mass (kg)	13.81 ± 3.52	6.80–20.60
Body water (kg)	37.97 ± 9.49	23.20–52.30
Lean mass (kg)	51.91 ± 12.98	31.80–71.70
Waist circumference (cm)	75.75 ± 7.05	65.00–91.00
Hip circumference (cm)	100.78 ± 5.52	91.00–113.50
Waist/hip	0.75 ± 0.06	0.67–0.88
Waist/height	0.44 ± 0.03	0.40–0.53
Age (years)	25.00 ± 2.28	22.00–31.00
*Gender*	*n*	%
Female	9	56.3
Male	7	43.8

Abbreviations: x̄: mean; BMI, body mass index; BMR, basal metabolic rate; Max, maximum; Min, minimum; SD, standard deviation.

#### 3.3.2 Energy and Nutrient Intakes

The dietary intakes of healthy individuals on the day prior to the consumption of test or control bread during the 1st and 2nd weeks of the study were questioned, and their energy and nutrient intakes were evaluated (Table 4). The median values of energy, macro‐ and micronutrient intakes, as well as antioxidant intakes, in healthy individuals during the 1st and 2nd weeks did not differ statistically (*p* > 0.05). The antioxidant intakes of healthy individuals (TEAC, TRAP, TOTAL ORAC, FRAP1, FRAP2, FRAP3, HORAC) are presented in Table 4.

**TABLE 4:** Energy, macronutrient, and antioxidant intakes of healthy individuals.

Variables	1st week			2nd week			*p*
	Median	Q1	Q3	Median	Q1	Q3	
Energy (kcal)	1442.85	1018.60	1999.15	1293.35	998.05	2110.80	0.642
Protein (g)	64.65	42.25	80.35	53.20	46.95	99.60	0.717
Fat (g)	62.15	51.40	100.30	55.85	38.55	112.15	0.836
Carbonhydrate (g)	129.55	105.65	190.30	144.30	120.75	203.50	0.379
Fiber (g)	14.25	11.15	23.80	16.10	7.55	25.55	0.679
TEAC (μmol TE/g)	63.60	32.80	78.55	64.70	25.60	93.65	0.796
TRAP (μmol/L or μmol TE/g)	53.20	39.45	116.70	87.65	28.50	141.35	0.717
TOTAL_ORAC (μmol/L or μmol TE/g)	10860.00	7192.45	13595.00	9410.65	6213.50	12593.50	0.877
FRAP1 (μmol Fe^2^+/g)	7.20	6.25	9.80	8.65	5.80	14.35	0.140
FRAP2 (μmol Fe^2^+/g)	2.05	1.05	3.15	1.45	1.05	2.50	0.641
FRAP3 (μmol Fe^2^+/g)	231.90	109.95	436.45	301.20	187.20	723.00	0.379
HORAC (μmol TE/g)	10641.00	6787.85	13301.50	9047.80	5964.90	12243.00	0.836

*Note:* Wilcoxon signed‐rank test; Q1: First quartile, Q3: Third quartile.

Abbreviations: FRAP, Ferric Reducing Antioxidant Power; HORAC, Hydroxyl Radical Antioxidant Capacity; ORAC, Oxygen Radical Absorbance Capacity; TE, Trolox equivalent; TEAC, Trolox Equivalent Antioxidant Capacity; TRAP, Total Peroxyl Radical Trapping Antioxidant Parameter.

#### 3.3.3 Visual Analog Scales

In the Overall Appetite Scores of the Satiety Scale from the VAS, the test bread group showed statistically significantly higher values compared to the control bread group at 0 min (test: 53.75 [45.00–61.25] vs. control: 28.63 [26.75–30.25], *p* < 0.001) and at 30 min (test: 35.88 [32.50–40.13] vs. control: 27.38 [26.38–28.13], *p* < 0.001) (Table 5). No significant differences were observed between groups at other time points (*p* > 0.05). Regarding VAS Appetite Scores (desire for sweet, salty, sour, and fatty foods), no statistically significant differences were found between the groups (*p* > 0.05) (Table 5).

**TABLE 5:** Comparison of VAS satiety score and appetite score values between test and control bread consumption in healthy individuals.

Satiety scores	0. min	30. min	60. min	120. min	180. min
*VAS hunger*					
TB	5.50 (3.00–7.50)	4.00 (3.00–5.50)	5.00 (3.00–5.00)	6.00 (4.00–7.50)	7.50 (5.00–8.00)
CB	7.00 (4.50–8.50)	5.00 (3.50–6.00)	4.50 (3.00–5.00)	6.00 (3.50–6.50)	7.00 (4.50–8.00)
*p*	0.14	0.52	0.81	0.70	0.45
*VAS fullness*					
TB	3.00 (3.00–6.00)	6.00 (4.00–7.00)	6.50 (4.50–7.00)	4.50 (3.00–5.50)	3.00 (2.00–5.00)
CB	3.00 (1.00–4.50)	5.00 (4.00–7.50)	5.00 (4.50–8.00)	4.50 (3.00–7.00)	3.00 (2.00–6.00)
*p*	0.25	0.99	0.99	0.72	0.99
*VAS desire to eat*					
TB	6.50 (5.00–7.50)	6.00 (4.50–7.50)	6.00 (4.50–8.00)	6.00 (5.00–7.00)	6.50 (5.00–8.00)
CB	4.50 (2.50–8.00)	6.50 (4.50–7.50)	6.50 (5.00–8.00)	6.50 (5.00–7.50)	6.50 (3.00–8.00)
*p*	0.49	0.78	0.84	0.93	0.64
*VAS prospective consumption*					
TB	5.00 (3.00–6.00)	4.00 (2.00–5.00)	3.50 (2.50–5.00)	6.00 (4.50–7.00)	6.50 (5.00–7.00)
CB	6.50 (3.50–8.00)	3.50 (2.00–5.50)	4.50 (2.00–6.50)	4.50 (3.50–7.00)	6.00 (5.00–7.00)
*p*	0.21	0.64	0.54	0.42	0.87
*Overall appetite score*					
TB	53.75 (45.00–61.25)	35.88 (32.50–40.13)	27.00 (26.50–27.88)	28.00 (27.38–29.13)	28.75 (27.63–30.00)
CB	28.63 (26.75–30.25)	27.38 (26.38–28.13)	27.38 (26.13–27.88)	27.88 (26.75–28.88)	28.50 (27.25–29.88)
*p*	**< 0.001**	**< 0.001**	0.70	0.52	0.52

Appetite scores	0. min	30. min	60. min	120. min	180. min
*VAS sweet*					
TB	6.50 (4.00–8.00)	5.50 (3.00–8.50)	5.50 (4.00–7.50)	5.00 (4.00–8.00)	5.00 (4.00–8.00)
CB	6.00 (3.00–8.00)	7.50 (3.50–8.00)	6.50 (4.00–8.00)	7.00 (3.50–8.00)	6.00 (3.50–7.00)
*p*	0.84	0.70	0.62	0.78	0.78
*Vas salty*					
TB	7.00 (5.00–8.00)	6.50 (5.00–9.50)	6.00 (4.50–7.00)	5.00 (4.00–7.50)	5.00 (3.00–6.00)
CB	5.00 (3.50–7.00)	6.50 (4.50–8.50)	7.50 (3.00–9.00)	4.00 (2.50–8.50)	4.00 (3.00–7.50)
*p*	0.36	0.64	0.56	0.36	0.96
*Vas sour*					
TB	9.00 (8.00–10.00)	9.00 (8.00–10.00)	9.50 (8.00–10.00)	9.00 (8.00–10.00)	9.00 (7.00–10.00)
CB	9.00 (6.00–10.00)	8.50 (6.50–10.00)	9.00 (7.00–9.50)	8.50 (5.50–10.00)	8.00 (5.50–9.50)
*p*	0.10	0.47	0.31	0.52	0.45
*Vas fatty*					
TB	8.50 (5.00–10.00)	9.00 (5.00–10.00)	8.00 (4.00–10.00)	6.50 (4.00–10.00)	5.00 (3.00–9.50)
CB	7.00 (3.50–9.50)	6.50 (3.00–9.00)	6.00 (4.00–9.00)	5.50 (3.00–9.00)	4.50 (2.00–8.00)
*p*	0.80	0.34	0.59	0.70	0.54

*Note:* Med. (Q1–Q3) = Median (First Quartile‐Third Quartile), Mann Whitney *U* Test, Exact Test Result.

Abbreviations: CB, control bread; min, minute; TB, test bread; VAS, visual analogue scale.

#### 3.3.4 Biochemical Parameters

Similar glycemic responses were observed in the median BG values of healthy individuals following consumption of both the test and control breads, and no statistically significant differences were detected at any of the measurement times (0, 30, 60, 120, and 180 min) (*p* > 0.05) (Table 6). The area under the curve (AUC) values for the biochemical parameters also support this finding (test: 4482.75 vs. control: 4671.71; *p* = 0.69) (Table 7).

**TABLE 6:** Comparison of biochemical parameter values between test and control bread consumption in healthy individuals.

Biochemical parameters	0. min	30. min	60. min	120. min	180. min
*BG (mg/dL)*					
TB	90.40 (86.70–95.30)	123.40 (106.50–138.05)	105.80 (89.30–122.00)	89.25 (78.50–98.55)	82.60 (79.45–85.90)
CB	91.30 (85.70–97.45)	130.15 (108.35–135.85)	103.90 (97.95–133.20)	97.25 (88.45–108.05)	85.80 (75.95–93.15)
*p*	0.90	0.70	0.70	0.27	0.72
*TAS (mmol/L)*					
TB	1.41 (0.98–1.65)		1.12 (1.07–1.53)		1.10 (0.91–1.34)
CB	1.29 (0.93–1.52)		1.12 (0.90–1.51)		1.25 (0.90–1.36)
*p*	0.70		0.54		0.87
*TOS (μmol/L)*					
TB	4.31 (3.55–5.17)		5.85 (4.27–6.44)		5.40 (3.96–5.70)
CB	4.39 (3.46–5.57)		5.05 (3.71–7.14)		4.53 (3.43–5.31)
*p*	0.84		0.96		0.40

*Note:* Med. (Q1–Q3) = Median (First Quartile‐Third Quartile), Mann Whitney *U* Test, Exact Test Result.

Abbreviations: BG, blood glucose; CB, control bread; min, minute; TAS, total antioxidant status; TB, test bread; TOS, total oxidant status.

**TABLE 7:** Comparison of individual and mean AUC values (±SD) of biochemical parameters between test and control bread consumption.

Participant no	TAS (TB)	TAS (CB)	TOS (TB)	TOS (CB)	BG (TB)	BG (CB)
1	193.2	219.6	1213.2	1621.8	16770.0	21943.5
2	182.4	177.0	667.5	487.8	14451.0	16857.0
3	218.7	240.3	1003.5	1222.2	17578.5	16672.5
4	293.1	318.6	1046.1	943.2	20298.0	20680.5
5	266.4	301.5	890.7	570.0	18997.5	19654.5
6	190.5	247.5	1593.6	1032.6	16398.0	14962.5
7	256.2	347.7	849.6	725.1	19860.0	20403.0
8	284.4	244.5	765.9	838.8	18763.5	18009.0
9	267.0	237.9	944.4	803.7	15636.0	17091.0
10	203.1	144.9	700.5	772.5	15831.0	17568.0
11	168.3	131.7	855.3	1027.5	20877.0	18055.5
12	179.4	146.7	1052.4	841.5	23229.0	25063.5
13	178.2	160.8	1023.9	740.7	16674.0	20097.0
14	187.5	209.4	618.3	1340.4	18354.0	13923.0
15	267.3	231.6	1372.5	1093.5	16528.5	20454.0
16	194.1	157.2	913.8	900.6	16654.5	17554.5

	Mean ± SD	95% CI	Mean ± SD	95% CI	*W*	*p*
TAS	220.612 ± 43.574	197.394–243.831	219.806 ± 64.866	185.242–254.371	79	0.597
TOS	969.45 ± 257.407	832.288–1106.612	935.119 ± 287.477	781.933–1088.305	84	0.433
AKŞ	17931.281 ± 2292.99	16709.433–19153.129	18686.812 ± 2758.56	17216.88–20156.745	44	0.231

*Note:* Individual and summary AUC values (mean ± SD, 95% CI) for TAS, TOS, and BG after the test and control bread consumption. AUC values were calculated using the trapezoidal method. Wilcoxon signed‐rank test (*W*) statistics and *p*‐values are also provided. AUC values for TAS and TOS were calculated from measurements at 0, 60, and 180 min, while AUC values for BG were calculated from measurements at 0, 30, 60, 120, and 180 min. Data were obtained from 16 participants under both test and control bread consumption conditions.

Abbreviations: AUC, area under the curve; BG, blood glucose; CB, control bread; TAS, total antioxidant status; TB, test bread; TOS, total oxidant status.

No significant differences were observed between the test and control bread groups in terms of TAS and TOS levels, which are indicators of oxidative stress (*p* > 0.05) (Table 6). The area under the curve values also support this finding (test: 4482.75 vs. control: 4671.71; *p* = 0.69) (Table 7).

In Table 7, the AUC values obtained after consumption of the test and control breads were compared. No statistically significant differences were observed between the two groups in terms of BG, TAS, and TOS (*p* > 0.05).

Changes in BG, TAS, and TOS levels over a 180‐min period before (0 min) and after consumption of test and control breads by healthy individuals are presented in Figure 3. In Figure 3A, a similar postprandial glucose response was followed in both test and control breads; participants' glucose levels peaked at 30 min, and a gradual decline was observed. However, no statistically significant differences were observed between the groups at any time point (*p* > 0.05). According to Figure 3B, although a higher baseline TAS value was noted in the test group, this difference diminished over time, and TAS levels remained at similar levels between the groups at 60 and 180 min. No statistically significant differences were detected at any time point (*p* > 0.05). In Figure 3C, similar TOS curves were observed for both the test and control bread groups; although there was a slight increase observed at 60 min in the test bread group, this difference was not found statistically significant (*p* > 0.05).

## 4 Discussion

In this study, the effects of white bread formulations enriched with carob flour (CMP flour) and wheat germ on glycemic response, antioxidant capacity, and satiety were investigated. Chemical analyses showed that the test bread had significantly higher levels of total phenolic compounds, antioxidant capacity, and dietary fiber compared to the control bread. However, in a randomized, single‐blind, cross‐over controlled study conducted with 16 healthy volunteers, no significant difference was observed in BG, TAS, and TOS values after consumption of the test bread compared to the control bread. These findings suggest that despite its enriched nutrient composition, short‐term consumption of the test bread does not produce a measurable effect on glycemic response or oxidative balance.

The study included healthy individuals aged 19–35 with a BMI of 18.5–24.9 kg/m^2^. Excluding participants with acute or chronic diseases, those taking medication, or those with impaired glucose metabolism ensured that the results reflected physiological responses independent of external factors. This methodological approach provided an advantage in evaluating whether the obtained glycemic response, satiety, and antioxidant effects were solely attributable to the bread formulations. Similar studies with comparable sample criteria are also available in the literature (Wu et al. 2023; Gonzalez‐Anton et al. 2015).

When evaluated in terms of macronutrients, the test bread contains slightly higher amounts of fiber, protein, and fat than the control bread, while the amount of digestible carbohydrates is lower. These limited differences are consistent with studies showing that small macronutrient changes do not always produce measurable postprandial glycemic differences in healthy young adults (Meng et al. 2017). However, it is known that soluble fiber, in particular, reduces glucose fluctuations by delaying gastric emptying and slowing carbohydrate absorption (Tsitsou et al. 2023). Similarly, bread enriched with resistant starch or high‐amylose flour has been reported to reduce postprandial glucose and insulin responses, though the effect varies depending on type and dose (Belobrajdic et al. 2019; Bohl et al. 2025; Amaral et al. 2022). In our study, although the test bread had higher resistant starch and fiber content, the absolute amounts per serving may have been insufficient to produce a significant glycemic effect. Similarly, while the addition of wheat bran increases protein, fat, and antioxidant components, previous studies have shown that the glycemic response does not decrease significantly unless there are larger changes in macronutrients (Moreira‐Rosário et al. 2016). Carob flour polyphenols have been reported to inhibit α‐amylase and α‐glucosidase activities (Coe and Ryan 2016), but their acute effects on glycemic response are limited due to bioavailability and dose constraints in cereal‐based matrices (Ayua et al. 2021).

Chemical analyses also revealed that the test bread was significantly enriched in terms of fiber, phenolic compounds, antioxidant capacity, and resistant starch. These differences are mainly due to the addition of carob flour and wheat bran. In the literature, carob flour is defined as a functional component due to its high fiber and phenolic compound content (Papageorgiou et al. 2020; Ayaz et al. 2007), while wheat germ is reported to be rich in protein, fat, and antioxidant compounds and to improve the nutritional profile of bread (Brandolini and Hidalgo 2012; Türker et al. 1996). In this study, a high antioxidant capacity value of 40.18 mmol TE/kg was obtained as a result of the combined use of the two components. Considering that values reported in the literature typically range from 10 to 30 mmol TE/kg (Arranz and Saura Calixto 2010; Fardet et al. 2008), this difference is thought to stem from the type of components used, their concentration, extraction method, and measurement techniques (Muzolf‐Panek and Stuper‐Szablewska 2021).

Despite the positive nutritional profile of the test bread, there may be several reasons why no significant differences were observed in BK, TAS, and TOS values. First, the intervention was limited to a single, acute consumption; however, it is known that the effects of functional components such as dietary fiber, resistant starch, and polyphenols typically emerge with long‐term consumption (Watzl et al. 2002; Rashed et al. 2022). Second, the amounts of resistant starch and polyphenols provided in a 50 g bread portion are lower than the doses reported in studies where significant effects were observed (Belobrajdic et al. 2019; Amaral et al. 2022). Third, TAS and TOS values may exhibit high individual variability, and the emergence of systemic antioxidant effects may require a longer timeframe than the 180‐min measurement window used in our study (Takeuchi et al. 2024). Finally, despite the high phenolic content of the test bread, acute effects may be weakened due to the limited bioavailability and bioaccessibility of polyphenols in grain‐based matrices (Manach et al. 2004).

Unlike biochemical parameters, significant differences were found in satiety findings. The test bread provided higher subjective satiety scores compared to the control bread, especially in the early postprandial period (0 and 30 min). This finding is consistent with studies showing the satiety‐enhancing and energy intake‐reducing effects of bread enriched with fiber and protein (Lee et al. 2006; Amoah et al. 2021). Additionally, systematic reviews indicate that viscous fiber and resistant starch increase satiety in the early period, but their effects on hedonic food cravings (desire for sweet, salty, or fatty foods) are more inconsistent (Gonzalez‐Anton et al. 2015; Smith et al. 2012). In our study, no significant differences were found in appetite parameters (based on taste preferences), suggesting that bread fortification is effective in enhancing overall satiety but does not alter hedonic appetite dimensions.

Taken together, test bread enriched with carob flour and wheat bran had positive effects on satiety in the early postprandial period, but did not provide significant improvement in acute glycemic response or oxidative stress markers. The findings suggest that functional components may contribute to appetite regulation, but longer‐term interventions and higher doses are needed to achieve significant effects on metabolic parameters.

## 5 Limitations of the Study

This study has several limitations. Firstly, assessments were conducted only in the acute phase; thus the long‐term effects of the functional ingredients on glycemic control, oxidative stress, or appetite regulation could not be determined. Additionally, the study sample was limited to healthy young adults, and it remains unclear whether the findings are applicable to different age groups, individuals with chronic diseases, or those with insulin resistance. The relatively small sample size may have also limited the statistical power. Furthermore, since it was not possible to isolate the individual effects of the components used, it is challenging to distinctly attribute the observed effects to a specific ingredient.

## 6 Conclusion

The results of this study demonstrated that a sensory‐acceptable and nutrient‐enriched functional white bread formulation could positively contribute to early‐stage appetite management. The test bread, enriched with CMP flour and wheat germ, made a significant effect particularly on subjective satiety; however, no significant biochemical differences were observed in glycemic response or oxidative stress markers following short‐term consumption. These findings show that comprehensive evaluations are needed to better understand the potential effects of functional ingredients. In the future, there is a need for long‐term intervention studies with larger sample sizes, including diverse age groups and health profiles.

## Funding

This research received no specific grant from any funding agency in the public, commercial or not‐for‐profit sectors.

## Ethics Statement

This study was conducted in accordance with the principles of the Declaration of Helsinki and ethical approval was obtained from Mersin University Clinical Research Ethics Committee (Date: 17/08/2022, Decision No: 562).

## Consent

Written informed consent forms were obtained from participants in the study.

## Conflicts of Interest

The authors declare no conflicts of interest.

## Data Availability

The data that support the findings of this study are available on request from the corresponding author.
